# Supplementary material for: Probing weak chemical interactions of metal surface atoms with CO-terminated AFM tips identifies molecular adsorption sites
Source: Nat Commun. 2025 Aug 23;16:7874. doi: 10.1038/s41467-025-63159-x (PMC12374979; doi:10.1038/s41467-025-63159-x)
Supplement: Supplementary file 1 — Supplementary Information [file 41467_2025_63159_MOESM1_ESM.pdf]

Supplementary information for

Probing weak chemical interactions of metal surface atoms with CO-terminated AFM tips identifies molecular adsorption sites

Jalmar Tschakert[1,2], Qigang Zhong[1,2], Alexander Sekels[1,2], Pascal Henkel[2,3,4], Jannis Jung[2,3], K. Linus H. Pohl[2,3], Hermann A. Wegner[2,5], Doreen Mollenhauer[2,3,6,7,8*], André Schirmeisen[1,2], and Daniel Ebeling[1,2*]

[1]Institute of Applied Physics (IAP), Justus Liebig University Giessen, Heinrich-Buff-Ring 16, 35392 Giessen, Germany

[2]Center for Materials Research (LaMa), Justus Liebig University Giessen, Heinrich-Buff-Ring 16, 35392 Giessen, Germany

[3]Institute of Physical Chemistry, Justus Liebig University Giessen, Heinrich-Buff-Ring 17, 35392 Giessen, Germany

[4]Department of Applied Physics, Aalto University, PO Box 11000 00076 Aalto, Finland

[5]Institute of Organic Chemistry, Justus Liebig University Giessen, Heinrich-Buff-Ring 17, 35392 Giessen, Germany

[6]Institute for Technical and Environmental Chemistry, Friedrich Schiller University Jena, Philosophenweg 7a, 07743 Jena, Germany.

[7]Helmholtz Institute for Polymers in Energy Applications Jena (HIPOLE Jena), Lessingstrasse 12-14, 07743 Jena, Germany.

[8]Helmholtz-Zentrum Berlin für Materialien und Energie GmbH (HZB), Hahn-Meitner-Platz 1, 14109 Berlin, Germany.

*Email: Doreen.Mollenhauer@uni-jena.de, Daniel.Ebeling@ap.physik.uni-giessen.de

The supporting information contain:

- Supplementary figures 1-9
- Computational details
- Supplementary references

Supplementary note 1. Constant-height AFM images and spectroscopy curves on Cu(111) for different oscillation amplitudes

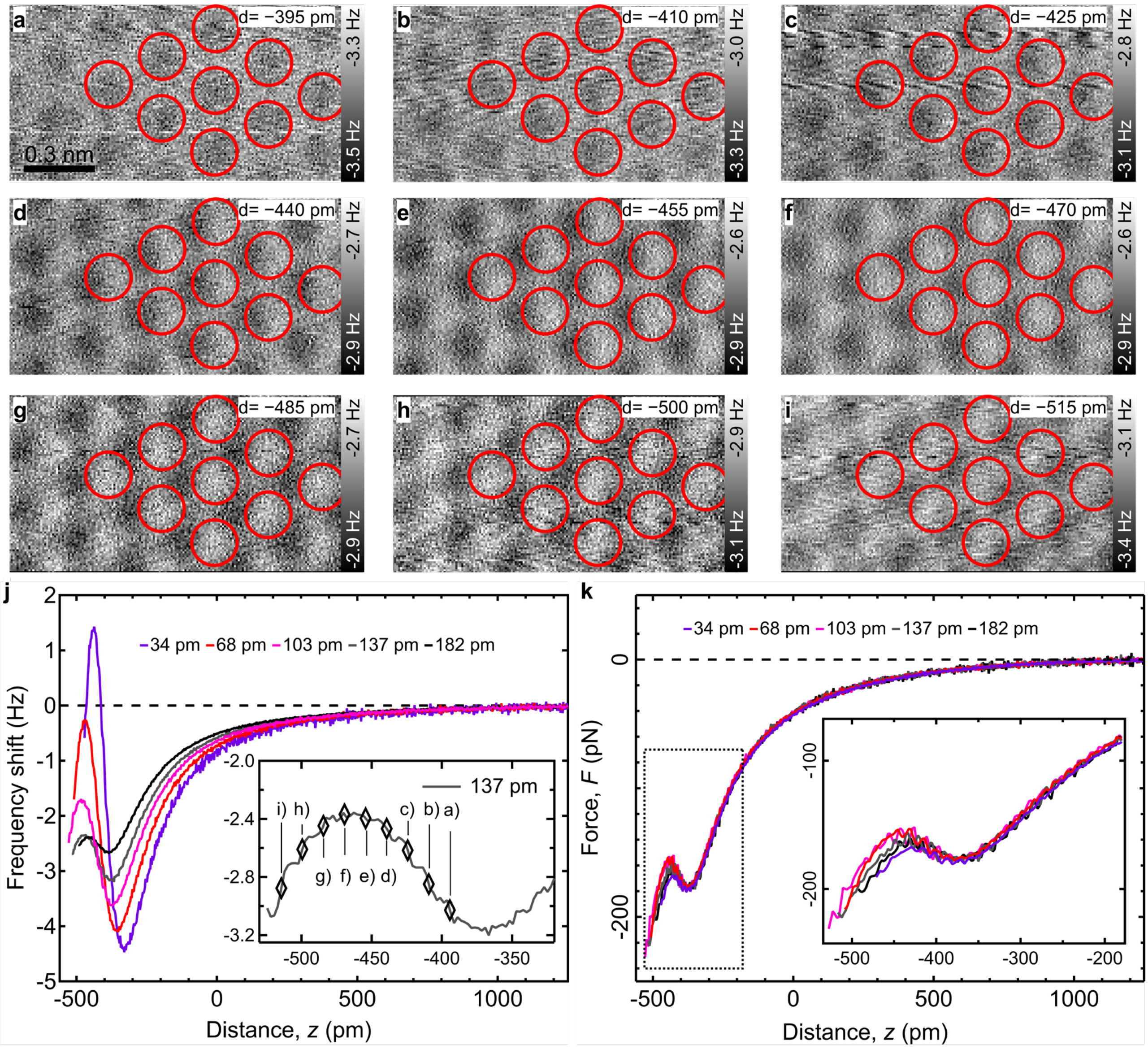

Supplementary Figure 1: Constant-height AFM images and spectroscopy curves on Cu(111) for different oscillation amplitudes. a-i Constant-height frequency shift AFM images of a Cu(111) surface measured with the same CO tip as in Figure 1 in the manuscript but with a higher oscillation amplitude of ≈ 137 pm. The average tip-surface distances are given with respect to the tunneling gap at I_{tunnel} = 10 pA and U_{sample} = 100 mV. The red circles in **a** are fitted to the dark regions and indicate the positions of the Cu atoms (top sites). These red circles were copied to **b-i**. **j,k** Frequency shift and force vs. tip-surface distance curves for five different oscillation amplitudes ranging from 34 pm (purple) to 182 pm (black). Qualitatively the frequency shift curves in **j** show a similar behavior. As expected the absolute frequency shifts depend strongly on the oscillation amplitude. [1] The measured force vs distance curves in **k** are independent of the oscillation amplitude, which demonstrates the reliability of the amplitude calibration. Insets: The inset in **j** indicates the positions of the constant-height images from **a-i**. The inset in **k** shows a zoom-in to the region indicated by the dashed box.

Supplementary note 2. Constant-height AFM images and spectroscopy curves on Cu(111) – series of 41 images at different tip-surface distances

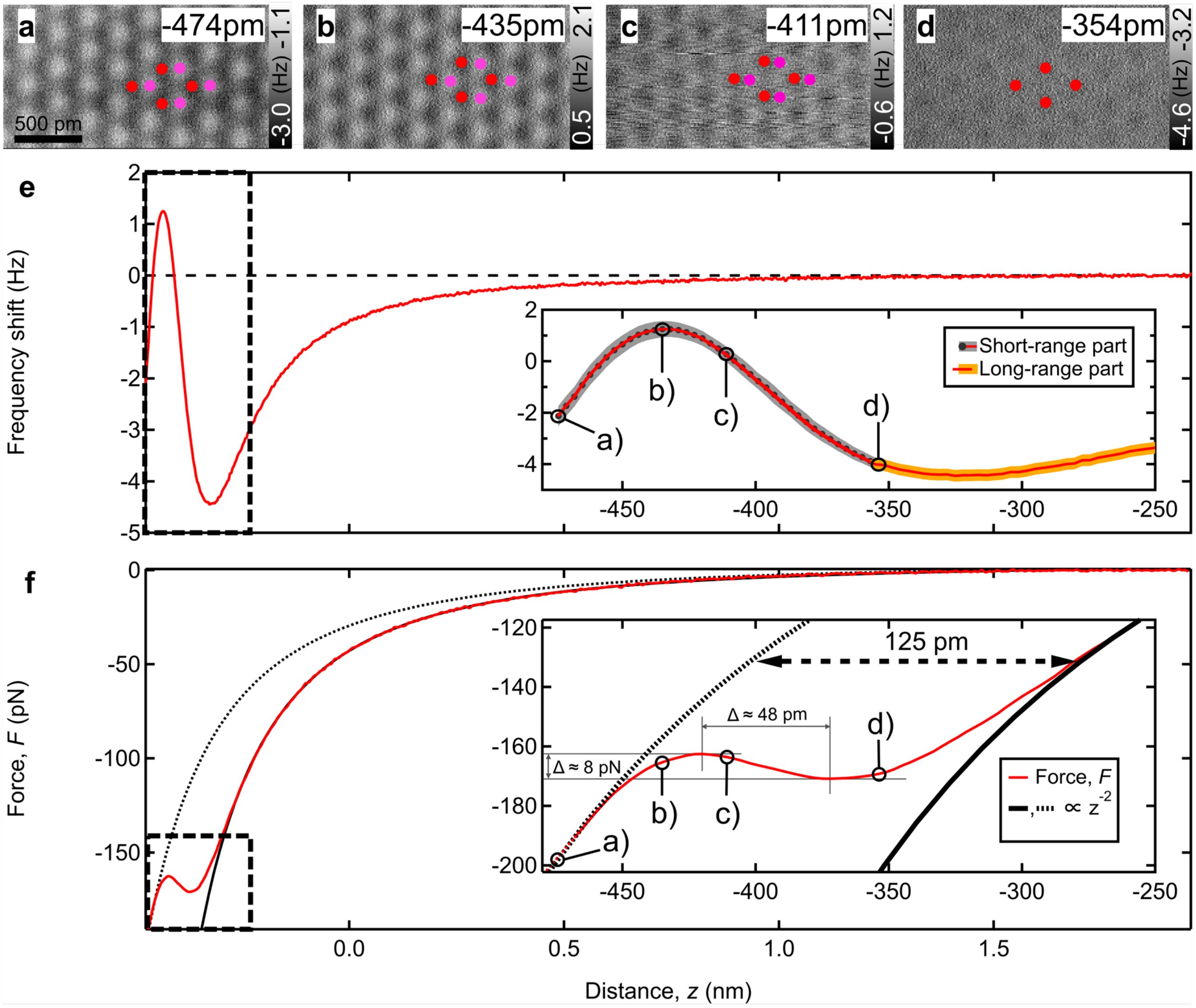

Supplementary Figure 2: Constant-height AFM images and spectroscopy curves on Cu(111) from a different measurement series. Here 41 constant-height frequency shift images were measured at different distances (data acquisition time ≈ 24 hours). The same CO tip and amplitude = 34 pm were used as for the "low drift measurement" shown in Fig. 1 in the manuscript. **a-d** Constant-height frequency shift AFM images of a Cu(111) surface measured at four different distances (taken from the 41 images). The average tip-surface distances are given with respect to the tunneling gap at I_{tunnel} = 10 pA and U_{sample} = 100 mV. The red dots indicate the dark images features, while the pink dots indicate the bright image features. At the largest imaging distance of -354 pm (**d**) only dark image features are visible. **e** Frequency shift vs tip-surface distance curve. The gray part (see inset) is taken from the 41 constant height images. The yellow part (see inset) is taken from a separate long range spectroscopy curve that was measured up to z = 2 nm. **f** Calculated force vs distance curve via Sader method.

Supplementary note 3. Constant-height AFM images of Ag(111)

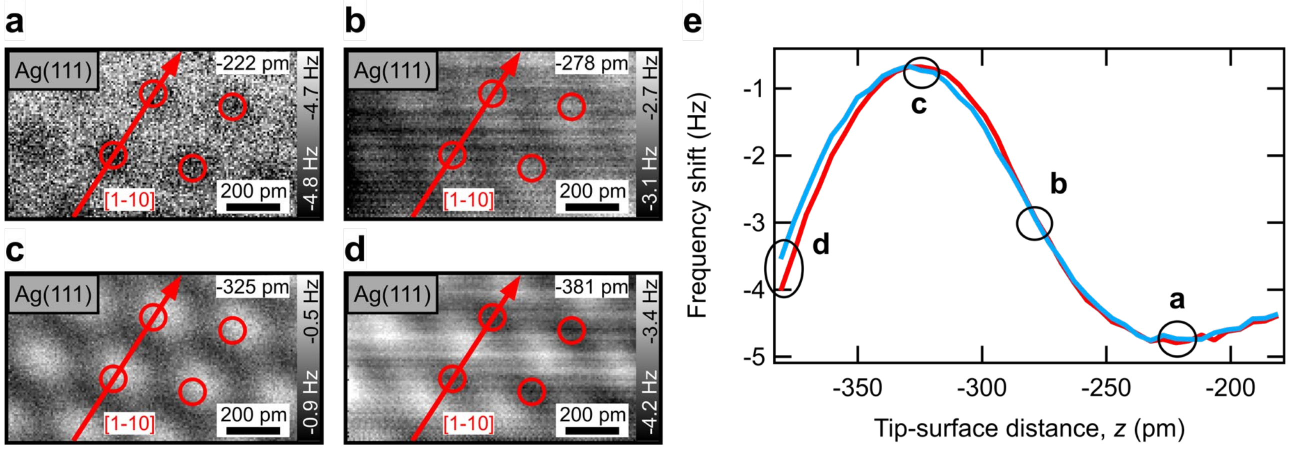

Supplementary Figure 3: Constant-height AFM images of Ag(111). a-d Constant-height frequency shift AFM images of a Ag(111) surface (image **a** is the same image as shown in Figure 2b in the manuscript). These images were taken from a 3D frequency shift vs distance measurement. The tip-surface distances are given with respect to the tunneling gap at I = 18 pA and U_{sample} = 7 mV and a vibration amplitude of 61 pm. **e** Frequency shift vs. distance curves taken above a top (red) and a bridge site (blue) (data also taken from 3D spectroscopy). The black markers indicate the tip-substrate distances for **a-d**.

Supplementary note 4. Constant-height AFM images of Au(111)

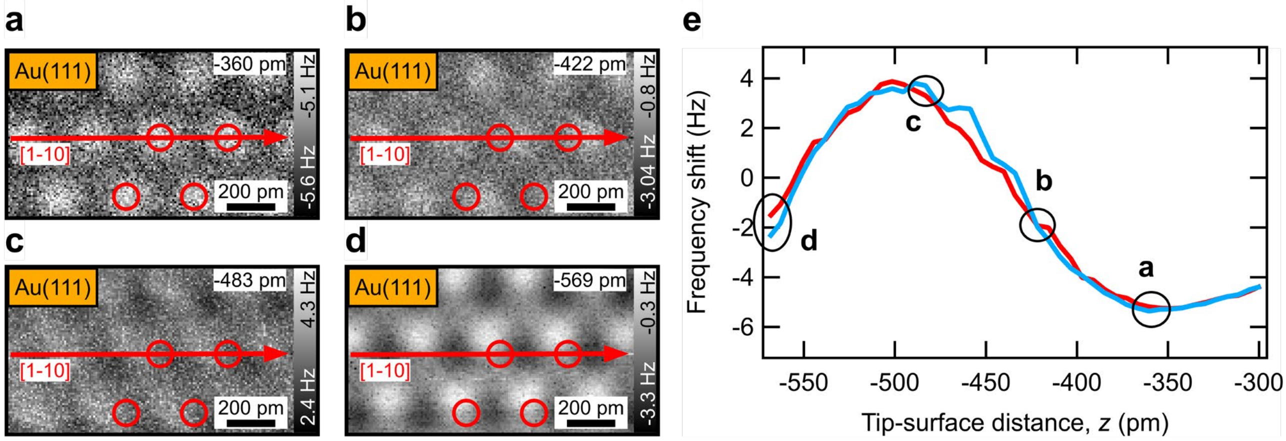

Supplementary Figure 4: Constant-height AFM images of Au(111). a-d Constant-height frequency shift AFM images of a Au(111) surface (image **a** is the same image as shown in Figure 2c in the manuscript). These images were taken from a 3D frequency shift vs distance measurement. The tip-surface distances are given with respect to the tunneling gap at I = 10 pA and U_{sample} = 100 mV and a vibration amplitude of 85 pm. **e** Frequency shift vs. distance curves taken above a top (red) and a bridge site (blue) (data also taken from 3D spectroscopy). The black markers indicate the tip-substrate distances for **a-d**.

Supplementary note 5. 2D force and potential energy maps of the Cu(111) and Au(111) surface

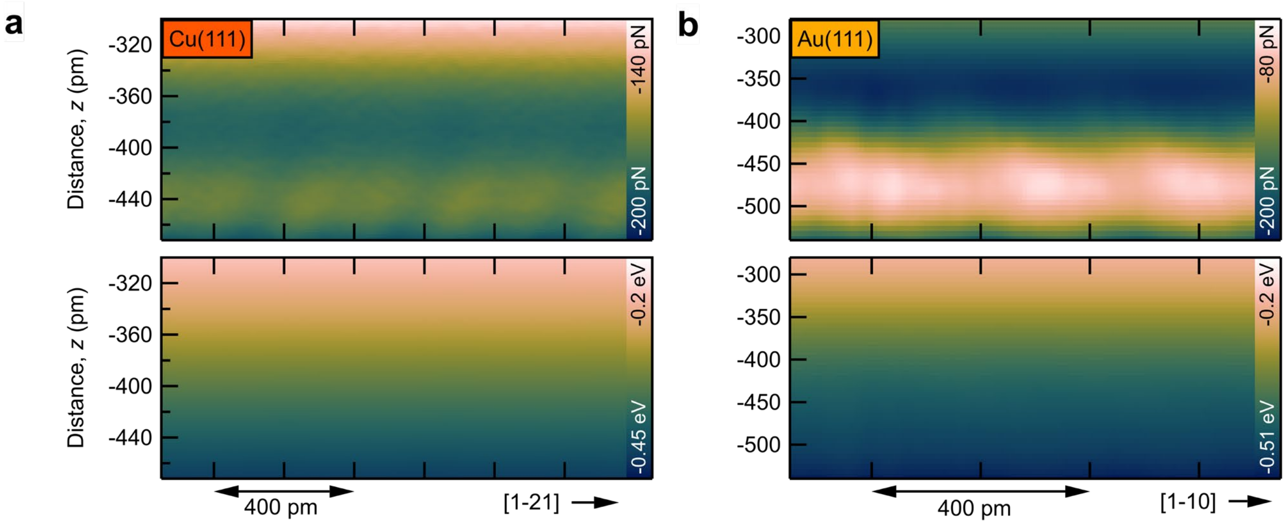

Supplementary Figure 5: 2D force and potential energy maps of Cu(111) and Au(111). a,b 2D force and potential energy vs distance maps calculated via the Sader method from the measured the frequency shift vs distance maps shown in Fig 2d and f, respectively.

Supplementary note 6. Modelling of the CO tip – metal surface system

Periodic unrestricted density functional theory calculations have been performed in order to study the interaction between the CO tip and the Cu(111)/Ag(111)/Au(111) surfaces, and for the analysis of the binding nature by using the Vienna ab initio simulation package (VASP) (version 5.4.1).[2, 3, 4, 5] We chose the Perdew-Burke-Ernzerhof (PBE)[6, 7] functional within the generalized gradient approximation (GGA)[8] in combination with the D3 dispersion correction with Becke-Johnson type damping function (BJ) developed by Grimme et al. [9, 10] The projector augmented wave method (PAW) was used in combination with a plane wave basis set with the kinetic energy cutoff set to 425 eV [11, 12] (deviation to higher kinetic energy cutoff <1 ‱). To sample the Brillouin zone, a Γ-centered $3 \times 3 \times 1$ k-point grid was used for the structural optimization[13] and a $7 \times 7 \times 1$ grid in combination with the tetrahedron method with Blöchl correction for the energy optimization[14] (deviation to higher k-points <3 ‱). The Gaussian smearing method with a σ value of 0.2 eV was utilized to describe the electronic occupancies. In addition, the conjugate gradient method was chosen with a convergence criterion of 10^{-4} eV and a threshold of 10^{-5} eV for the total energy.

In order to model the CO tip - metal surface interaction, we used the experimental lattice constants of 3.61 Å for Cu, 4.09 Å for Ag and 4.08 Å for Au to model the hexagonal Cu(111)/Ag(111)/Au(111) surfaces.[15] Thus, we constructed $p(6 \times 6)$ surface supercells with three layers (16 atoms/layer), whereby the bottom layer was fixed to represent the bulk phase. The two other layers were freely relaxed in all three dimensions, representing the surface. In order to avoid the periodic interaction of the surfaces, a vacuum of 12 layers (resulting in a total vacuum height of ~25 Å) was added to the slab models. In addition, a dipole correction was applied in $\langle 001 \rangle$ surface direction.[16] We modeled the CO tip by using four metal atoms representing the tip. The four metal atoms were arranged pyramidal - three atoms form the base and the fourth the peak - in agreement with the study by Huber et al.[17] The three metal atoms forming the tip base were fixed to simulate the tip bulk phase and the CO molecule was placed at the peak metal atom. The peak metal atom and the CO molecule, in contrast, were freely relaxed in all three dimensions. To calculate the force vs. distance curves, the height of the whole tip (including the 4 metal atoms and the CO molecule) was adapted; where the distance was measured between the metal surface and the oxygen atom.

Furthermore, while calculating the force-distance curves, we observed that the perfectly symmetrical AFM tip model suppresses bending of the CO molecule at the apex at short distances. To address this, we repeated the calculations with the tip slightly tilted out of the *ab* plane, making it no longer perfectly perpendicular to the surface (see Suppl. Fig. 6). Specifically, the entire tip - consisting of four metal atoms and the CO molecule - was pitched by 10° around the *y* axis, with the O atom remaining centered above the four adsorption sites (top, bridge, fcc, and hcp) prior to relaxation (see Suppl. Fig. 6). This symmetry breaking allows the CO molecule to bend at short distances during relaxation. At larger tip-surface distances, the resulting force-distance curves closely resemble those obtained with the perfectly perpendicular tip. At smaller distances, however, the CO molecule bends as expected, in line with experimental observations, see dotted lines in Suppl. Fig. 6.

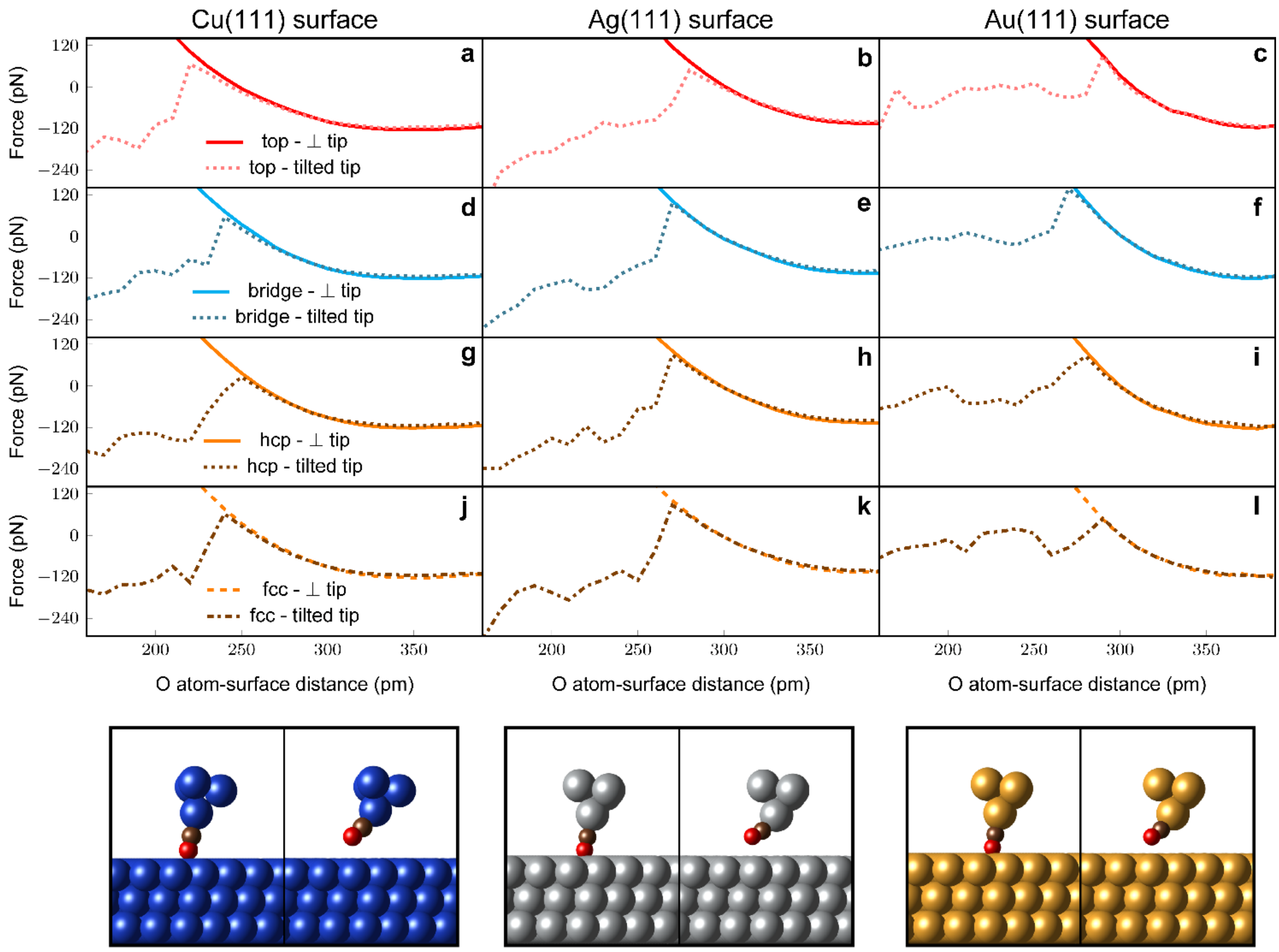

Supplementary Figure 6: Force vs. distance curves for Cu(111), Ag(111), and Au(111) using using a slightly tilted tip calculated at PBE-D3(BJ)/pw (PAW) level of theory . The curves above top, bridge, and hollow sites (fcc and hcp) are colored in red, blue, and orange, respectively. Solid lines correspond to the perfectly symmetric tip (results identical to Fig. 4 in the manuscript), while dotted lines represent the tilted tip configuration (see bottom for a representation of the tiled tips). Tip representations for Cu(111), Ag(111) and Au(111) surfaces are shown at the bottom-left:prior structural optimization and bottom-right: after relaxation. The O atom-surface distance refers to the pre-relaxation value in order to maintain a consistent distance reference across configurations.

As the CO molecule passes through the force minimum, the forces initially increase - similar to the behavior observed with the perfectly symmetric system - until the CO molecule begins to bend, at which point the forces decrease again. At short distances, the calculated forces exhibit significantly more fluctuations compared to those at larger distances. This is primarily due to the CO molecule displacing not only along the *z*-axis but also within the *ab* plane during bending. Consequently, the orientation of the CO molecule varies slightly between different tip-surface distances. In this low-force regime, even minor variations in orientation between data points can lead to noticeable differences in the calculated forces. To mitigate this, statistical averaging over multiple orientations at the same distance would be necessary. Despite these variations, the bending behavior of the CO molecule at short distances clearly reproduces the experimental trends observed on Cu(111), Ag(111), and Au(111) surfaces.

Sun et al. demonstrated that the interaction force between a CO-functionalized tip and a metal surface is predominantly determined by the CO-surface interaction, with only a minor contribution from the metallic tip cluster. [18] To verify that our results are not influenced by the tip cluster size - particularly given that the forces at the top, bridge, fcc, and hcp sites amount to only a few pN - we repeated the simulations using an extended

three-layer tip. In this model, the original tip (see Suppl. Fig. 8) was augmented with an additional layer of six metal atoms. The modeling approach remained unchanged: the apex metal atom, the CO molecule, and the second layer were fully relaxed in all spatial dimensions, while the six atoms of the third layer - forming the base of the tip - were fixed to mimic the bulk-like structure of the tip, see Suppl. Fig. 7.

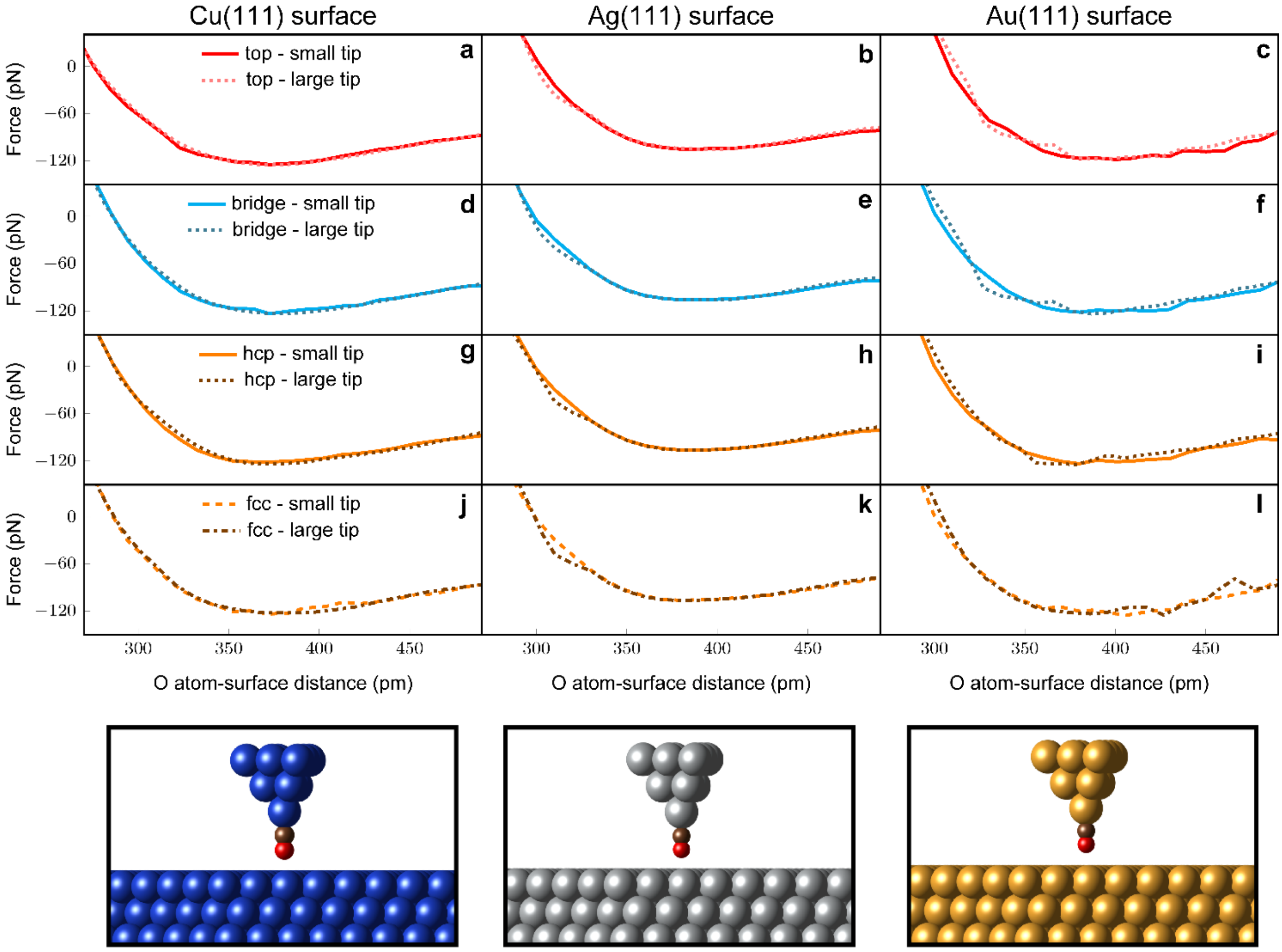

Supplementary Figure 7: Force vs. distance curves for Cu(111), Ag(111), and Au(111) using a larger three-layer tip calculated at PBE-D3(BJ)/pw (PAW) level of theory. The curves above top, bridge, and hollow sites (fcc and hcp) are colored in red, blue, and orange, respectively. Solid lines represent the smaller tip (results identical to Fig. 4 in manuscript), while dotted lines correspond to the larger tip (see bottom for a representation of the larger tip constructed out of three metal atom layers). The force vs distance curves for Cu(111), Ag(111), and Au(111) calculated with the larger tip were shifted by +10 pN, +12 pN and +15 pN, respectively, to align each curve at the corresponding top site, facilitating better comparison.

Overall, the results clearly indicate that increasing the tip size leads only to a systematic offset in the calculated forces, while the relative ordering of the interacting forces at the top, bridge, fcc, and hcp sites on all three metal surfaces remains unaltered. This confirms that the size of the metal cluster does not affect the observed trends.

The LOBSTER program[19, 20, 21, 22] (version 4.0.0) was used to analyze the binding nature of the interaction between the oxygen from the CO tip and a Cu(111)/Ag(111)/Au(111) surface atom [see Suppl. Fig. 8] by means of (projected and integrated) crystal orbital overlap population (COOP). The binding nature has been studied in the energy minimum of the energy-distance curves [see Fig. 4 in manuscript and Suppl. Fig. 8] for the top position. The used structures were optimized in advance by VASP. The *pbeVaspFit2015*[22] basis set was used for the projection of the plane waves onto localized orbitals. The following basis functions were used: Cu (4*s*,

4*p* & 3*d*), Ag (5*s*, 5*p* & 4*d*), Au (6*s*, 6*p* & 5*d*), C (2*s* & 2*p*) and O (2*s* & 2*p*). The charged spilling values - indicating the information loss during the projection - are about 1 % for each surface-CO tip system.

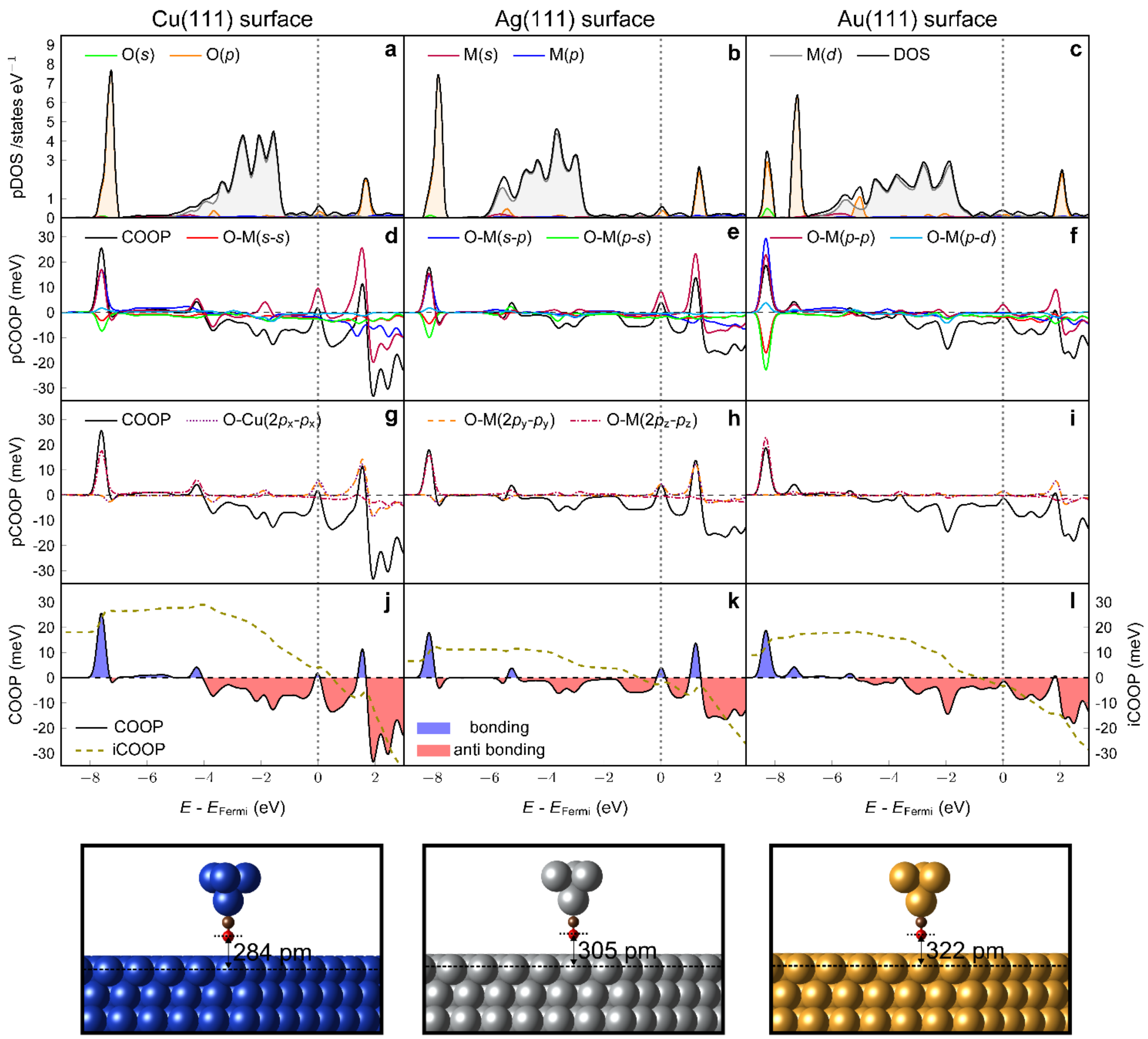

Supplementary Figure 8: Calculated pDOS, pCOOP, and integrated pCOOP for a CO tip above Cu(111), Ag(111), and Au(111) surface on top position calculated at PBE-D3(BJ)/pw (PAW) level of theory. The tip-surface distances, i.e., the distance between the oxygen of the CO tip and the metal surface plane, were 284 pm, 305 pm, and 322 pm, respectively, which correspond to the locations of the potential minima. The CO tip was placed above a top site in each case. The total density of states and COOP are plotted as black lines, while the colored lines give the contributions of the *s*- and *p*-states of the oxygen atom and the *s*-, *p*-, and *d*-states of the metal atoms below the oxygen.

To better understand the origin of the observed image contrasts, we have studied in detail the binding situation between the CO tip and the metal surface. In particular, we investigated the interaction between the oxygen atom of the CO tip and the top position metal atom of the metal surface Specifically, we considered the interaction of the O(2*s*) and O(2*p*) orbitals with the M(*s*), M(*p*) and M(*d*) orbitals of the top position metal atom in the range between -9 eV and +3 eV at the Fermi level. Note that the covalent binding contributions are very small and that large distances between the CO tip and the metal atom of the surface of 284 pm on Cu(111),

305 pm on Ag(111), and 322 pm on Au(111) are present. The pDOS shows that the O-M_{top} (M = Cu, Ag, Au) interaction is mainly governed by the overlap of the O(2*p*) and M(*p*) orbital and the COOP analysis of each orbital contribution [see **d** to **f**] supports this, see purple line in d) to f). For all three systems, we find that the O(2*p)*-M(*p)* contribution has a small binding contribution at E_F. This contribution at E_F results largely from the interaction of O(2p_x)-M(p_x) orbitals and O(2p_y)-M(p_y) orbitals. In addition, there is another stronger binding O(2*p)*-M(*p)* contribution at ≈ -8 eV, which is mainly determined by the interaction of O(2p_z) with M(p_z).

The iCOOP value of 4 meV including all states up to E_F indicates an overall binding nature for the CO tip Cu(111) interaction and an anti-binding nature for Ag(111) (-2 meV) and for Au(111) (-3 meV).

Supplementary note 7. Image contrast above an adsorbed organic molecule

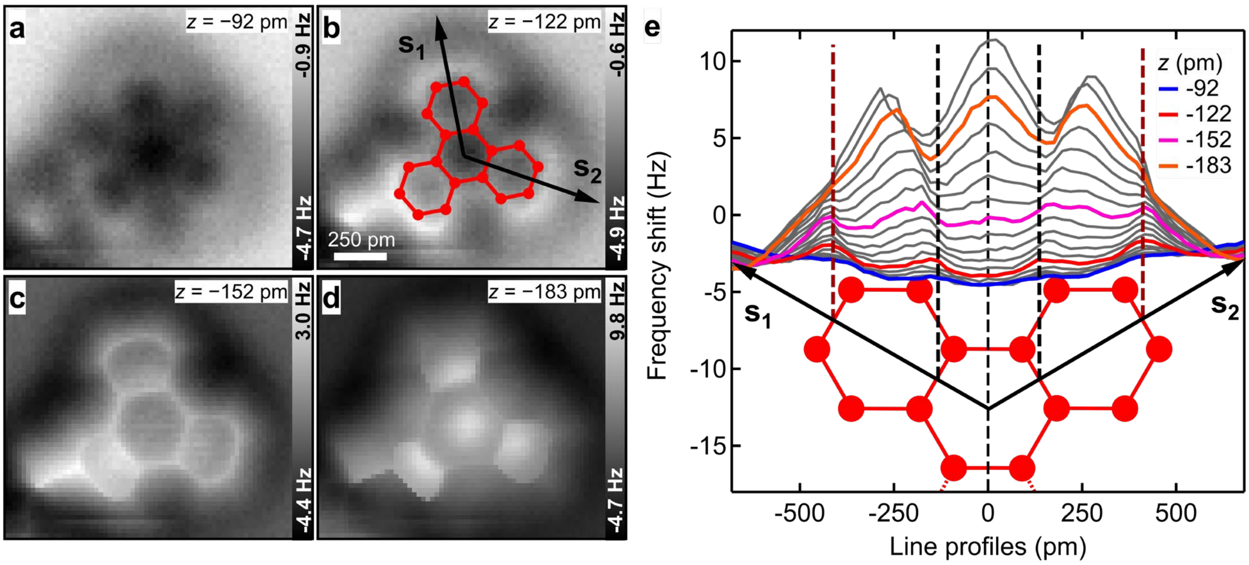

Supplementary Figure 9: 3D constant-height frequency shift vs. distance spectroscopy dataset of 2-triphenylenecarbaldehyde on Cu(111). a-d Four *xy*-slices at different average tip-surface distances. The *z*-values are given with respect to the tunneling gap at U_{sample} = 100 mV and I = 10 pA. The vibration amplitude was ≈ 51 pm and the quality factor was ≈ 22700. The fitted positions of the carbon atoms are shown as red markers in **b**. **e** Individual line profiles along the path indicated by the two black arrows S1 and S2 in **b**. The profiles that correspond to the tip-surface distances in **a-d** are colored in blue, red, pink, and orange, respectively, while the other profiles are shown in gray.

The images in Suppl. Fig. 9a-d clearly reveal the image distortions and contrast inversion at relatively small tip-surface distances that are typical when imaging adsorbed organic molecules with CO-terminated tips. The line profiles in Suppl. Fig. 9e reveal that the positions where the carbon-carbon bonds appear systematically shift with decreasing tip-surface distance. However, the imaging distance does not influence the overall position of the molecule, which is identical for all images of the 3D data set. Therefore, when determining adsorption positions of molecules it needs to be taken into account that the atomic features of the surface are shifting in lateral direction with decreasing imaging distances, while the position of the molecule is rather unaffected.

Supplementary references

1. Hölscher H, Allers W, Schwarz UD, Schwarz A, Wiesendanger R. Determination of Tip-Sample Interaction Potentials by Dynamic Force Spectroscopy. *Phys Rev Lett* **83**, 4780–4783 (1999).

2. Kresse G, Hafner J. Ab initio molecular dynamics for liquid metals. *Phys Rev B* **47**, 558–561 (1993).

3. Kresse G, Hafner J. Ab initio molecular-dynamics simulation of the liquid-metal--amorphous-semiconductor transition in germanium. *Phys Rev B* **49**, 14251–14269 (1994).

4. Kresse G, Furthmüller J. Efficiency of ab-initio total energy calculations for metals and semiconductors using a plane-wave basis set. *Computational Materials Science* **6**, 15–50 (1996).

5. Kresse G, Furthmüller J. Efficient iterative schemes for ab initio total-energy calculations using a plane-wave basis set. *Phys Rev B* **54**, 11169–11186 (1996).

6. Perdew JP, Burke K, Ernzerhof M. Generalized Gradient Approximation Made Simple. *Phys Rev Lett* **77**, 3865–3868 (1996).

7. Perdew JP, Ernzerhof M, Burke K. Rationale for mixing exact exchange with density functional approximations. *The Journal of Chemical Physics* **105**, 9982–9985 (1996).

8. Langreth DC, Perdew JP. Theory of nonuniform electronic systems. I. Analysis of the gradient approximation and a generalization that works. *Phys Rev B* **21**, 5469–5493 (1980).

9. Grimme S, Antony J, Ehrlich S, Krieg H. A consistent and accurate ab initio parametrization of density functional dispersion correction (DFT-D) for the 94 elements H-Pu. *The Journal of Chemical Physics* **132**, 154104 (2010).

10. Grimme S, Ehrlich S, Goerigk L. Effect of the damping function in dispersion corrected density functional theory. *Journal of Computational Chemistry* **32**, 1456–1465 (2011).

11. Kresse G, Joubert D. From ultrasoft pseudopotentials to the projector augmented-wave method. *Phys Rev B* **59**, 1758–1775 (1999).

12. Blöchl PE. Projector augmented-wave method. *Phys Rev B* **50**, 17953–17979 (1994).

13. Monkhorst HJ, Pack JD. Special points for Brillouin-zone integrations. *Phys Rev B* **13**, 5188–5192 (1976).

14. Blöchl PE, Jepsen O, Andersen OK. Improved tetrahedron method for Brillouin-zone integrations. *Phys Rev B* **49**, 16223–16233 (1994).

15. Kittel C. *Introduction to solid state physics*. Wiley: Hoboken, NJ (2005).

16. Neugebauer J, Scheffler M. Adsorbate-substrate and adsorbate-adsorbate interactions of Na and K adlayers on Al(111). *Phys Rev B* **46**, 16067–16080 (1992).

17. Huber F, Berwanger J, Polesya S, Mankovsky S, Ebert H, Giessibl FJ. Chemical bond formation showing a transition from physisorption to chemisorption. *Science* **366**, 235 (2019).

18. Sun Z, Boneschanscher MP, Swart I, Vanmaekelbergh D, Liljeroth P. Quantitative Atomic Force Microscopy with Carbon Monoxide Terminated Tips. *Phys Rev Lett* **106**, (2011).

19. Deringer VL, Tchougréeff AL, Dronskowski R. Crystal Orbital Hamilton Population (COHP) Analysis As Projected from Plane-Wave Basis Sets. *J Phys Chem A* **115**, 5461–5466 (2011).

20. Maintz S, Deringer VL, Tchougréeff AL, Dronskowski R. Analytic projection from plane-wave and PAW wavefunctions and application to chemical-bonding analysis in solids. *Journal of Computational Chemistry* **34**, 2557–2567 (2013).

21. Dronskowski R, Blochl PE. Crystal Orbital Hamilton Populations (Cohp) - Energy-Resolved Visualization of Chemical Bonding in Solids Based on Density-Functional Calculations. *J Phys Chem-Us* **97**, 8617–8624 (1993).

22. Maintz S, Deringer VL, Tchougreeff AL, Dronskowski R. LOBSTER: A tool to extract chemical bonding from plane-wave based DFT. *J Comput Chem* **37**, 1030–1035 (2016).
